# Basis of a National Dental Association

**Published:** 1859-06

**Authors:** J. G. Hamill

**Affiliations:** Lancaster, Ohio.


### BASIS OF A NATIONAL DENTAL ASSOCIATION.

BY J. G. HAMILL.

IN the April number of the *Dental News Letter*, we have presented a "Basis of a National Dental Association." While we do not object to the basis, or to the establishment of a society upon it; yet we can not consent for such a society to take the place of the American Dental Convention. The suggester of this "basis," says of the American Dental Convention, that "it was intended, indeed, only to subserve the exigencies of the period, and to prepare the way for a more permanent and useful association." That it was the offshoot of the necessities of the times, we will admit; but that it was only intended to subserve a transient purpose—we don't believe it. A few, such as "Junius," might have thought so, but the profession at large never thought so. We care but little how "ephemeral" it may be considered. We always looked upon each meeting of the Convention as complete in itself, and not necessarily bound by all its antecedent ways of doing things.—The absence of "constitution, laws, and conditions for membership," is, in our opinion, the great redeeming point of the society. It is that which makes it national. In fact we do not believe that any society embracing the dental profession of America, can be organized on any plan that will be more permanent, than that of the American Convention.

Man is, by nature, an imperfect being, consequently every thing that emanates from him, is stamped with his imperfections. He is so blinded by a naturally corrupt heart, that he can not always show the same mercy to others, that he would like to have shown to himself under similar circumstances. Hence, the necessity of having a National Convention entirely free from conditions. One of the most striking facts in the universe is, that there are not two persons on the earth whose features are alike, and it is none the less true that no two minds think alike. Why, then, should it be thought possible, (to say nothing of the practicability of the thing,) for an association, with constitution and laws, to enlist the support of even a majority of the profession. It never has been the case. It never will be the case. A constitution and laws may operate very well for local societies, and can be submitted to with a degree of complacency; but it won't do for national societies. That was the very rock the American Society of Dental Surgeons split upon. Its laws were such that its own founders and warmest supporters were compelled to break over and ignore them. Hence, its death, and the American Dental Convention in its place.

As to the usefulness of the American Dental Convention, it has been beyond all expectation. It has done what no other society could have done, and we think the whole secret of its success lies in the fact that it was founded upon a basis coextensive with the limits of our country, and tolerant as the wants of erring man require. This basis is commendable because it seeks to benefit, not only a few, who are already as high on the ladder as any have gone; but also all those who have not enjoyed the best advantages for improvement. It is commendable, also, because it is calculated to call out the experience of all, whether old or young.

"Remarks must not become discussional on personal methods; but each in speaking should confine himself wholly to the explanation of his own method."

Such was the language of (Dr. Rich) the first President of the first Convention. It is a noble hearted expression. It implies volumes; and should be the controlling motto of every convention that pretends to be national. All men are not alike constituted. Some are timid; some are bold; some are sarcastic; some are *over* zealous; hence the necessity of conducting a convention in accordance with the above sentiment. It meets the case of every one, and none need stand back in dread of the older and more experienced. As it is the drops of water that make up the ocean, so it is the mites of truth that appear in the experience of the many, that fills our great professional store-house. We have received more information from reading the free descriptions of modes of personal practice as reported from the American Dental Convention, than from any other one source. The beauty of the thing is, we have the advantage of comparing our own practice with that of so many others; and by combining the best points of them all, with that of our own, we have a bundle of experience as near perfect as our profession has yet attained to.

But "Junius" tells us that "its day of usefulness, however, is apparently past; its absurdities censured with just cause; losing the support of its best men; its end seems near at hand." Truly, this is a sad picture; but we don't believe it is true. Judging from the benefits derived from the last meeting, we have no right to conclude that its usefulness is past. We have not heard its best men say they were going to leave it, so we have no right to believe it is losing them. Its end does not seem any nearer to us than it did this time last year. It met last summer; doubtless, it will meet this summer, also. As to exposing and censuring its

"absurdities," of course, that is all right. It is not reasonable to suppose that two or three hundred men should talk together for two or three days, and tell nothing that was not false, in theory and practice. And when such is the case, they ought to be censured. They ought to be pitched into, and reviewed *sharply*. If they are honest, it will do them good, if they are not so, why, it makes no difference. A man may think he is saying some smart things, and those that listen to him may think so, too; but when they become sifted of their flowery language, and the substance reported, without the speaker's life-giving accompaniments, they may appear very flat. It may be, this is the reason why some of the members of the Convention are disposed to quarrel with the reports so much. They ought to remember that it is the "rubbing together of the millstones that brings out the flour."

We have shown some of the good the American Dental Convention has done, and given some reasons why it has been so much more productive of good than any other national society of dentists. Now, that it may still accomplish the same, or a greater amount of good, it is only necessary that the profession take the same interest in it that it has done. This, we firmly believe, it will do.

"Junius" gives it as his suggestion, that a representative association will have more "stability and character, and be deprived of any approach to a clique." Now, it is our opinion that such a society would be a clique in spite of everything. Local societies are all cliques, (I guess they don't claim to be anything else,) and their representatives would necessarily partake of the same. He also rests a claim for a representative association in that "it would exercise an influence and wield a power, possessing all the force of a government; deriving its just powers from the consent of the governed, it would rest its claim of authority on the equitable principle that in representative bodies the majority must rule." Now we are democratic enough to appreciate the doctrine that all governments derive their power from the consent of the governed, and that the majority must rule; but where is the justice of the comparison between governmental law and professional law. Governmental law is fixed—is arbitrary; is laid down in "black and white," and every one is compelled, by the fear of punishment, to shape his actions in accordance with it. But the very moment you set up a society with "laws, and conditions of membership," you *exclude* the majority. There is no fear of any punishment except banishment, and that, by a majority, will be self-imposed. Professional laws *pretend* to be liberal—pretend to hold the man up, and lead his mind out to renewed exertions for the promotion and exaltation of his profession. All dentists can not live up to the same rule of practice. It is impossible, from the nature of the organs they have to treat and the obscurity of the causes of their disease; and, also, the modification of those diseases and their causes, as found in different latitudes and climates. Take, for instance, caries of the teeth. Can a man be found that can tell exactly what agents produce white decay in the torrid, temperate or frigid zones? No. The thing can't be done. In fact, we *know* but little *about* the causes of caries, and some other diseases of the teeth; and as to their *treatment*, we are nothing but *empirics*.

How, then, in view of these facts, are we to have a *national* association, hedged in by a constitution and laws, that will not exclude some who are as justly entitled to a place in it as others? How, too, if this representative "basis" be adopted, are so much practical experience and results to be arrived at? Shall every local society write out its views upon all subjects, and direct its delegates to say it off in *school boy* fashion, to the Convention? Certainly, that won't do. If the representative plan be adopted, every delegate will go up there, (if he don't stay away,) and give his *own opinion* on all the subjects that may be discussed, and that will be the end of it. If the present plan of convention be continued, we will have a larger meeting, consequently more experimental knowledge, and a fuller and more unrestrained expression of opinion. So, you see, we have planted ourself upon the broad platform of "the greatest good to the greatest number." That such a spirit should always characterize our *National Dental Societies*, is my most ardent wish.

Lancaster, Ohio, April 13, 1859.

